# Neuroscience of consciousness in the locked‐in syndrome: Prognostic and diagnostic review

**DOI:** 10.1002/ibra.12077

**Published:** 2022-11-07

**Authors:** Berenika Maciejewicz

**Affiliations:** ^1^ Department of Biomedical Engineering Einstein Medical Institute North Palm Beach Florida USA

**Keywords:** brain injury, cognition, disorders of consciousness, locked‐in syndrome, neuroscience of consciousness

## Abstract

The neurological illness known as a locked‐in syndrome is brought on by damage to the brainstem, usually as a consequence of a stroke. It is characterized by total paralysis with intact consciousness and cognitive capacity. The subjective experiences of people with locked‐in syndrome are poorly understood. Presently, there is no systematic evaluation developed to describe them. The most compelling resources come from individuals’ own words; however, only a small fraction of these accounts have been explored. When it comes to bioethics, locked‐in syndrome protocols are almost completely absent. Investigations on how people with this condition feel about their sense of continuity are of importance. Utilizing the locked‐in syndrome to pose questions on embodied cognition and levels of consciousness could serve as a lens through which to examine problems in the phenomenology of neuroparalysis and communication. Care and quality of patients’ lives might be improved by an effort to understand this condition better, and ontological questions like “what makes a person a person?,” “what makes a person appear in continuity?,” and “what are the dynamics of embodiment and intersubjectivity?” might be better explored through that lens. This article aims to explore some biomedical factors that contribute to locked‐in syndrome and offers some prognostic and diagnostic recommendations for this rare condition.

## Graphical abstract

Locked‐in syndrome classification after acute brain injury. Patients are often initially in a come before gradually regaining consciousness. The motor recovery chart shows full recovery in 7% of the cases. No recovery comprises 21% of all cases.

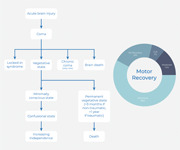

## 1 INTRODUCTION

Locked‐in syndrome (LIS) happens in persons with bilateral ventral central nervous system injuries. One of the most common triggers of this condition is a stroke in the pontine region. Other common causes include encephalitis, brain tumors, malignant neoplasms, head trauma, and central forebrain myelinolysis.^1^ The patient is a quadriplegic due to injuries to both ventral pons corticospinal circuits. As a result of trauma to the corticobulbar tracts, a person loses the ability to express themselves verbally and via facial expressions. The syndrome is sometimes abbreviated as LIS and is very rare. Some of the patients who have LIS can have satisfying lives, can communicate with others through eye movements and externally provided tools, and in some unusual cases even find employment.^2^ The syndrome causes the patient's voluntary muscles to become paralyzed, except for the muscles that control the vertical movement of the eyes.^3^ People who have LIS are awake, aware, and have their regular cognitive skills. This means that they can think and reason, but they are unable to talk, move, or express facial emotions. Although those who have LIS can hear, their main form of communication is often through the use of blinking, deliberate eye movements, or both.^4^ They also have the option of using a wide variety of assistive technologies in the course of their conversation. Injury to the pons, which is a portion of the brainstem, is what causes the LIS. People who suffer from the many forms of LIS tend to not feel pain, although this is not a given. Patients who experience the form of LIS described as incomplete could still experience pain and perhaps other physical sensations as well.^5^ Due to the significant possibility of misdiagnosis and underreporting of cases, researchers have a difficult time gaining an accurate estimate of the yearly number of individuals affected. LIS as a condition poses key questions on cognition and levels of consciousness in the field of neuroscience. As such, it might serve as an important lens through which to investigate dilemmas in the areas of neuroparalysis, communication, and detection of levels of awareness in this disorder of consciousness.

## 2 METHODOLOGY

The primary type of information used in the investigation of LIS was qualitative data. Qualitative data was more useful for this research and evaluation than quantitative data that nearly does not exist in this still newly emerging subject of study. Patients with LIS may interact with researchers, so physicians can use a battery of diagnostic tools to look into the disease, determine what causes it, and rule out other neurological disorders. Examples of such examinations are computed tomography (CT) scans or magnetic resonance imaging (MRI): The effects of an MRI or a CT scan may reveal whether or not the pons or other brain regions have been damaged. Cerebral angiography is a diagnostic procedure used to find out the presence or absence of a blood clot in the brainstem or brain arteries. The electrical activity of a patient's brain may be measured using an electroencephalogram (EEG). It may help doctors tell whether a subject has LIS by gauging whether or not their brain activity and sleep–wake cycles are atypical.

## 3 RESEARCH

Individuals who suffer from LIS are deficient in certain neurotransmitters, which is the reason why they are unable to talk or move their limbs or facial muscles. As a result of the brainstem injuries that cause the LIS, the majority of affected individuals can communicate with the outside world via eye blinking and motions of the eye.^6^ There are three subtypes of LIS, all of which have the characteristic of inhibiting all movements apart from vertical eye blinking and movements. Additionally, some imperfect voluntary mobility may still be apparent in a patient, such as in the head or fingers. Patients of most LIS types, however, are rendered entirely motionless, sometimes including their eyes.^7^ As a result of advancements in central nervous system interface technology, patients who fall into this group are now able to manage their environment by using synthetic voice and eye‐controlled machine communication to command machines, browse the internet, and write.

In the first, classical form of this condition, although individuals are unable to blink, look up, and preserve all mental abilities, full immobility or a loss of muscle movement is one of the defining characteristics. Hearing is also preserved. In the incomplete form of the condition, the sole difference between this iteration of LIS and the more conventional one is that it may be possible to restore some functions related to movement and sensations in certain parts of the body. In the total immobility form of this illness, one loses the ability to move their eyes or any part of their body, and cognitive functions of the brain may also be impaired. An EEG is a type of test that looks at brain waves. It may be able to show if a person with this type of condition still has cognitive abilities left.

When there is significant damage to the brainstem, the LIS patient will become entirely paralyzed, except for their eyes, which they may be able to open and shut at their own will.^8^ The fact that many people with LIS begin in a coma or a vegetative state before recovering from it, possibly without anyone knowing as they are not able to communicate their alertness to their doctors or families, adds a layer of complexity to the challenge of getting an accurate diagnosis.^9^ Although life with LIS looks dreadful, there is rising awareness of this uncommon condition. National Organization for Rare Disorders^10^ is one of these organizations that posts and promotes information on therapeutic and treatment options for those who have LIS. In many instances, the symptoms that are present at the numerous other states of the disorders of consciousness overlap with one another, and there is always the chance that some of these symptoms may vary over the course of time. When clinicians have an accurate picture of their vegetative patients’ state of consciousness and awareness, they are better equipped to diagnose their patients, successfully provide sufficient treatments, and make decisions for their end‐of‐life care.^11^

Aphasia and quadriplegia are some of the characteristics of locked‐in syndrome. Quadriplegia is the inability to move or utilize any of one's limbs. Paralyzed individuals might be able to connect with others via the use of coded signals because eye blinking and movement are rarely impaired by paralysis.^12^ There is a chance that some individuals may recover mobility in their extraocular muscles as well as their facial muscles.^13^ However they are unable to create sounds at their own will, regardless of whether or not their vocal cords are paralyzed.

LIS is the opposite of a protracted vegetative state, which is initiated by damage to higher brain areas while leaving lower brainstem regions uninjured.^14^ An injury to the pons is the most common and studied reason for the syndrome to emerge. The symptoms might also come from poisoning, most often resulting from the bite of a krait or one of many other neurotoxic poisonous bites. Paralysis affects not only the patient's skeletal muscles but also their breathing muscles; however, artificial respiration can keep the patient alive.^15^ LIS caused by impairment to the pons may be brought on by a variety of different health conditions and accidents. The utmost shared cause of this condition is a stroke, either ischemic or hemorrhagic, which may impair the corticospinal, corticopontine, or corticobulbar circuits of the brainstem.^16^ A hemorrhagic stroke is a term given to the condition that occurs when arterial blood abruptly seeps into the brain. Other causes may involve tumors that are cancerous and may be found on the pons, amyotrophic lateral sclerosis, or Guillain–Barré syndrome.^17^

**Figure 1:**
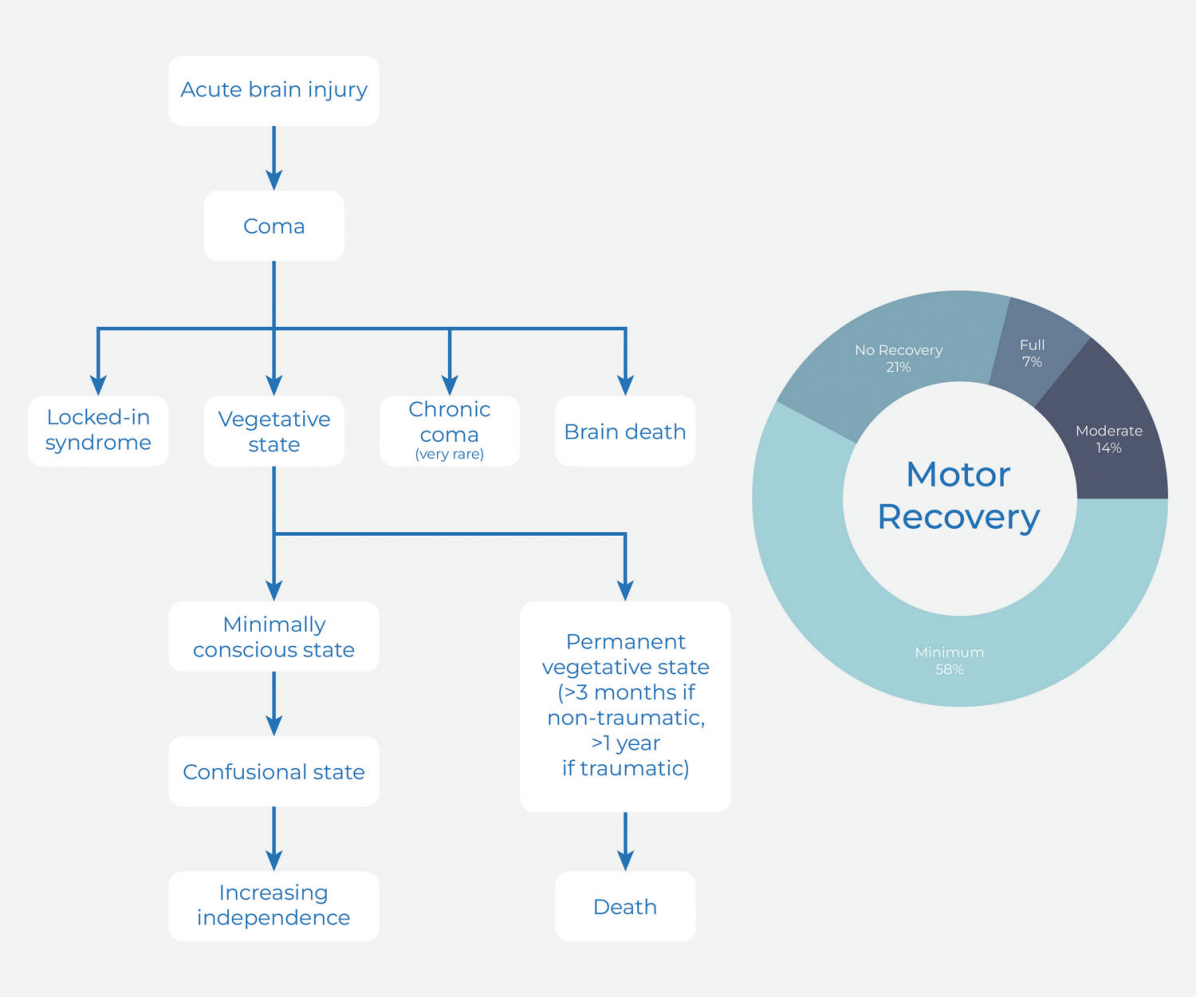
Locked‐in syndrome classification and motor recovery [Color figure can be viewed at wileyonlinelibrary.com]

Locked‐in syndrome may be difficult to diagnose. Patients do not display conventional motor responses like pain avoidance. They do not demonstrate muscular movements like withdrawal from painful stimuli, which are used to evaluate attentiveness; thus, medical professionals may make the mistaken assumption that these patients are unresponsive (Figure 1). As a remedy, testing may entail requesting specific actions from the patient, like a wink or a vertical eye gaze. Individuals also need the ability to start typical motor responses. Performing an EEG test may show patterns of sleep and wakefulness, which produces some evidence that the person is not unconscious but only paralyzed.

There is presently no treatment that is generally recognized for this condition, let alone a cure for it. With the use of neuromuscular electrical stimulation (NMES), some patients can regain a part of their muscle function. In most other situations, the treatment of symptoms is the standard practice.^18^ People with LIS may be able to connect with their environment better if they are offered eye‐tracking technologies like Dasher.^19^ Recommended tests to confirm a diagnosis of locked‐in syndrome, ascertain its etiology, and rule out confounding factors are scans using MRI or CT. This is to determine which regions of the brain sustained the damage. Next, cerebral angiography would help in determining if there is a presence of a blood clot in the brainstem or any arteries in the brain. An electroencephalogram is a device that is used to measure the electrical impulses that occur in human brains. Determining if brain activity and sleep–wake cycles are abnormal helps to diagnose LIS as well. Next, evoked potentials are a type of neuroimaging that can detect changes in the brain and spinal cord electrical activity in response to particular inputs from the outside. The findings of these evaluations are used by medical specialists to establish how much of one's brain and brainstem have been damaged. The health of one's muscles and nerves can be assessed with the use of a test called electromyography. The results of this test might assist medical experts in ruling out the possibility of just muscle and nerve injury.^20^ Pontine myelinolysis can be found with blood tests, like a full metabolic panel. Examination of cerebrospinal fluid (CSF) is also recommended. CSF in the brain and spinal cord can be tested to see if the symptoms are caused by an infection or an autoimmune disease.

Treatment of the underlying causes and prevention of consequences, if feasible, are the only options for those suffering from LIS. LIS is treated with a mix of psychosocial approaches, such as teaching people how to talk to their physicians and families, and if the extent of damage allows for regaining some motor functions, a supportive physical therapy might also be recommended. Treatment to aid breathing and eating must start right away. To help those with LIS breathe, doctors will often perform a tracheotomy and insert a tube into the patient's airway via a tiny incision in the throat. Many people with LIS have a gastrostomy tube surgically injected into their abdomen instead. Avoiding complications like pneumonia, urinary tract infections, and thrombosis that may arise from inactivity by engaging in basic physical therapy is a viable option. Some degree of voluntary movement may be regained in rare cases.^21^

Communication training is recommended as well. Patients with LIS may benefit from speech therapists’ guidance in using eye movements and blinking to express themselves. Each individual has their preferred means of conveying ideas. For instance, one may communicate “yes” by looking up and “no” by looking down, or vice versa. Words and phrases may be formed by people with LIS by having them indicate individual letters of the alphabet while another person vocally lists the letters. Patients with LIS can also communicate and use websites and online communications equipment like the infrared movement of the eye detectors and PC voice prostheses, in addition to using coded visual stimuli for interacting with others.

## 4 CONCLUSION

In conclusion, LIS is a relatively rare condition. It occurs in certain people with quadriplegia and decreased horizontal eye movements due to bilateral ventral pontine lesions. Patients may blink and move their eyes up and down because the supranuclear oculomotor pathways are more deeply embedded in the brain's dorsal cortex. Restrictions to horizontal eye movement occur because of damage to the nucleus and fibers of cranial nerve VI. If the reticular formation has not been compromised, mental integrity has been preserved. If the dorsal supranuclear oculomotor pathways were spared, the patient's ability to blink and perform vertical eye movements may be intact. The only way the patient can communicate is by blinking his or her eyes; all other movements are frozen. The development of new brain–computer interfaces may lead to the discovery of more seamless future therapies. Currently, medical professionals and neuroscientists are successful in eliciting yes‐or‐no responses from patients who otherwise are entirely immobilized to express themselves by movement or speed by utilizing a computer interface and specialized programs. There is also some ongoing research to develop new means for individuals who are confined to communicate with their relatives and physicians by using their unaffected sense of smell. Solutions using implants that could interpret brain activity are currently undergoing testing in Europe.^21^ The applications of diagnostic and prognostic protocols covered in this article will continue to face a multitude of logistical, theoretical, ethical, and psychological challenges. However, due to the significance of their clinical and scientific implications, the efforts of the research in the area of neuroscience of consciousness remain of paramount importance.

## AUTHOR CONTRIBUTIONS

The listed authors contributed to the conceptual framework and revised the manuscript and were responsible for the design, operation, data collection, data curation, data statistics, graphic production, writing, and revision.

## CONFLICT OF INTEREST

The author declares no conflict of interest.

## TRANSPARENCY STATEMENT

All the authors affirm that this manuscript is an honest, accurate, and transparent account of the study being reported; that no important aspects of the study have been omitted; and that any discrepancies from the study as planned (and, if relevant, registered) have been explained.

## ETHICS STATEMENT

The research was conducted in accordance with the principles embodied in the Declaration of Helsinki and in accordance with local statutory requirements. All participants or their legal guardians gave written informed consent to participate in the study.

## Data Availability

The data used to support this study is available from the corresponding author upon request.
